# Supplementary material for: Single-Domain Antibodies Represent Novel Alternatives to Monoclonal Antibodies as Targeting Agents against the Human Papillomavirus 16 E6 Protein
Source: Int J Mol Sci. 2019 Apr 28;20(9):2088. doi: 10.3390/ijms20092088 (PMC6539864; doi:10.3390/ijms20092088)
Supplement: Supplementary file 1 [file ijms-20-02088-s001.pdf]

International Journal of *Molecular Sciences*

MDPI

1. Supplementary Data (Methods)

1.1 Llama immunizations

Two llamas were immunized through services available at Cedarlane Laboratories (Burlington, ON, Canada), using the "short schedule" similarly described by Baral *et al.* [1]. The first (Immunization #1) was injected with 100 µg of His6-GenScript E6 + Freund's complete adjuvant on Day 0, followed by booster injections of 100 µg His6-GenScript E6 + Freund's incomplete adjuvant on Days 21, 28 and 35. The second (Immunization #2) was injected with 250 µg of His6MBP-4C/4S E6 + 250 µg of His6MBP-F47R 4C/4S E6 + Freund's complete adjuvant on Day 0, followed by booster injections of 250 µg His6MBP-4C/4S E6 + 250 µg His6MBP-F47R 4C/4S E6 + Freund's incomplete adjuvant on Days 21, 28 and 35. From both llamas, pre-immune blood was collected prior to the initial immunization as well as blood samples on Days 35 and 42, to allow confirmation of a successful immune response and to provide the lymphocytes subsequently used in the preparation of the VHH phage display libraries, as described below.

1.2 Serology

Confirmation of a successful immune response was first obtained by screening the total serum from blood drawn on Days 0 (pre-immune), 35 and 42 for antibodies which reacted with the recombinant E6 proteins by enzyme-linked immunosorbent assay (ELISA), as similarly described by Baral *et al.* [1], Hussack *et al.* [2], and Hussack *et al.* [3]. Briefly, 1 µg of His6-GenScript E6 diluted in PBS, or 0.5 µg His6MBP-4C/4S or His6MBP-F47R 4C/4S E6 diluted in storage buffer (50 mM Tris-HCl pH 6.8, 400 mM NaCl, 2 mM DTT) (100 µL/well) was coated in Nunc™ MaxiSorp™ 96-well plates (VWR; Mississauga, ON, Canada; Cat. #: CA10761-500) overnight at 4 °C. The wells were blocked with 5% (w/v) milk powder in PBS-T (PBS+0.05% (v/v) Tween 20) at 37 °C, prior to the application of serial serum dilutions for 1 h at room temperature. The wells were then washed with PBS-T and incubated with a goat anti-llama IgG + horseradish peroxidase (HRP) secondary antibody (Cedarlane Laboratories; Cat. #: A160-100P) diluted 1:10 000 in PBS for 1 h at room temperature. The wells were again washed with PBS-T and then incubated with 100 µL/well TMB substrate (Mandel Scientific; Guelph, ON, Canada; Cat. #: KP-50-76-00) for approximately 5 min. The reaction was stopped by the addition of 100 µL/well 1 M phosphoric acid and the absorbance read at 450 nm.

Day 0 and Day 42 total serum was then fractionated using protein A and protein G affinity chromatography as described by Baral *et al.* [1] and Hussack *et al.* [2] , with the addition of a second, pH 2.7 glycine buffer elution from the protein A column. Reducing SDS-PAGE was used to analyze the eluted G1 (IgG3 HCAb), G2 (IgG1 conventional antibody), A1 (IgG2a HCAb) and A2 (IgG2b/c HCAb) serum fractions, prior to confirmation of a positive HCAb immune response using ELISA, as similarly described above. In this instance, wells were coated with either 0.75 µg of His6-GenScript E6 diluted in PBS or a mix of 0.5 µg His6MBP-4C/4S E6 + 0.5 µg His6MBP-F47R 4C/4S E6 diluted in PBS (100 µL/well).

1.3 Construction of VHH phage display libraries

Two VHH phage display libraries were constructed, one corresponding to each immunization (*i.e.*, Library #1: His6-GenScript E6 immunization and Library #2: His6MBP-E6 immunization). For each library, RNA was extracted from ~1.0 x 10^8 frozen Day 42 lymphocytes using the PureLink™ RNA Mini Kit (Thermo Fisher Scientific; Mississauga, ON, Canada; Cat. #: 12183018A) and first-strand cDNA synthesis was performed using Superscript™ VILO™ Master Mix (Thermo Fisher Scientific; Cat. #: 11755050) with ~2 µg template RNA per reaction (total number of reactions: 4). PCR amplification of the VHH DNA was then completed, using the primers and procedures similarly described by Baral *et al.* [1]. Briefly, cDNA was first amplified in 50 µL reactions consisting of 3 µL

cDNA, 5 pmol MJ1-3 primer mixture, 5 pmol of either CH2FORTA4 or CH2B3-F primer, 1 μL 10 mM dNTPs, 0.5 μL Platinum™ *Taq* DNA Polymerase (Thermo Fisher Scientific; Cat. #: 10966026), 1.5 μL *Taq* kit 50 mM $MgCl_2$, 5 μL *Taq* kit 10x buffer, and 38 μL nuclease free H_2O using thermocycler parameters of 94 °C for 5 min, 35 cycles of 95 °C for 30 s, 56 °C for 45 s and 72 °C for 60 s, and a final extension of 72 °C for 10 min. A total of 8 PCR reactions were performed and pooled for each primer pair. PCR products of ~600 bp (for reactions containing CH2FORTA4 primer) and ~650 bp (for reactions containing CH2B3-F primer) were extracted from 1.5% agarose gels, purified and then re-amplified in 50 μL reactions consisting of ~20 ng template DNA, 10 pmol MJ7 primer, 10 pmol of MJ8 primer, 1 μL 10 mM dNTPs, 0.5 μL Platinum™ *Taq* DNA Polymerase, 1.5 μL *Taq* kit 50 mM $MgCl_2$, 5 μL *Taq* kit 10x buffer, and nuclease free H_2O using thermocycler parameters of 94 °C for 5 min, 35 cycles of 95 °C for 30 s, 58 °C for 45 s and 72 °C for 60 s, and a final extension of 72 °C for 10 min. A total of 16 PCR reactions were performed and pooled for each DNA template. The ~450 bp PCR products were purified, digested with *Sfi*I restriction enzyme, and purified again. The inserts were then ligated into pMED1 phagemid vector [4] which had also been digested with *Sfi*I restriction enzyme (with the addition of *Xho*I and *Pst*I restriction enzymes to reduce vector self-ligation) and the ligated vectors purified.

Finally, 20 x 50 μL aliquots of TG1 *E. coli* (Agilent Technologies; Santa Clara, CA, USA; Cat. #: 200123) were each transformed with 5 μL (~350 ng) ligated pMED1 using a BioRad® Gene Pulser electroporation device, titered and grown overnight at 37 C, 250 rpm in 2xYT supplemented with 100 μg/mL ampicillin and 2% (w/v) glucose, as described by Baral *et al.* [1]and Hussack *et al.* [2]. The following morning, the bacteria were pelleted, resuspended in 20 mL 2xYT supplemented with 100 μg/mL ampicillin and 2% (w/v) glucose + 20 mL 50% (v/v) glycerol, and stored in 1 mL aliquots at -80 °C. Colonies on the titer plates were counted as well as analyzed using colony-PCR (15 μL reactions consisting of 1.5 pmol -96gIII primer, 1.5 pmol M13RP primer, 0.4 μL 10 mM dNTPs, 0.15μL GenScript *Taq* DNA polymerase (GenScript; Cat. #: E00007), 1.5 μL Taq kit 10x buffer, 12.7 μL nuclease free H_2O, and a single colony pick using thermocycler parameters of 94 °C for 5 min, 35 cycles of 95 °C for 30 s, 55 °C for 30 s and 72 °C for 45 s, and a final extension of 72 °C for 10 min), to determine the total and functional library sizes. Two aliquots of library cells were thawed, infected with ~7.2 x 10^{11} plaque-forming units M13KO7 helper phage (New England BioLabs; Ipswich, MA, USA; Cat. #: N0315S), grown overnight, and the phage particles harvested from the culture supernatant and titered, as described by Baral *et al.* [1], Hussack *et al.* [2] and Hussack *et al.* [3], to create the input library phage for panning. Aliquots of ~100-200 μL input phage in PBS were kept at -80 °C for long-term storage.

1.4 Subtractive panning

The screening of both VHH phage display libraries for binders to the recombinant HPV16 E6 proteins was completed using panning techniques [5] routinely employed by us and others [1-3,6-8]. In the first round, two Immuno™ Breakable Module MaxiSorp™ wells (Thermo Fisher Scientific; Cat. #: 12-565-134) were coated with 10 μg of maltose binding protein (MBP), one was coated with a mix of 5 μg His6MBP-4C/4S E6 + 5 μg His6MBP-F47R 4C/4S E6, and one was coated with PBS (100 μL/well; antigens diluted in PBS) overnight at 4 °C. All wells were then blocked with 2% milk powder in PBS for 1 h at 37 °C. To subtract the MBP binders, 100 μL Library #1 input phage (~1 x 10^{12} phage) + 100 μL 4% milk powder in PBS were added to each of the two blocked, MBP-coated wells and incubated for 1 h at room temperature. Phage from one MBP-coated well were then transferred to the blocked, His6MBP-E6-coated well and phage from the other MBP-coated well were transferred to the blocked, "PBS-coated" well. Phage were incubated for 30 min at room temperature. The same procedure was completed in parallel using Library #2 input phage. Following washing with PBS-T, bound phages were collected in two sequential elutions: 1) 100 mM triethylamine neutralized with 1 M Tris-HCl pH 7.4 and 2) 100 mM glycine pH 2.0 neutralized with 2 M Tris base, and both elutions pooled. Exponentially growing TG1 cells were infected with the eluted phage and an aliquot taken for titration. The remaining infected cells were then superinfected with ~20x excess M13KO7 helper phage, grown overnight at 32 °C, and the amplified phage purified, as described by Baral *et al.* [1],

Hussack *et al*. [2] and Hussack *et al*. [3], to create input phage for the second round of panning. Here, amplified phages were quantified spectrophotometrically using the Antibody Design™ Laboratories Phage Concentration Calculator (http://www.abdesignlabs.com/technical-resources/phage-calculator/) which employs a formula based on the measurements by [9]:

$$\frac{(A_{269} - A_{320}) \cdot 6\times10^{16}}{number\ of\ bases/virion} = virions/mL$$

A second round of panning was completed as described above, except that each subtraction well was coated with an increased amount (20 µg) of MBP, each target antigen well was coated with a decreased amount (2.5 µg His6MBP-4C/4S E6 + 2.5 µg His6MBP-F47R 4C/4S E6) of E6 proteins, and the blocking buffer switched to SuperBlock™ (Thermo Fisher Scientific; Cat. #: PI37580), to increase selective pressure for higher affinity binders and prevent enrichment of VHHs with affinity for milk powder or MBP.

1.5 Phage ELISA

To examine progress after each round of panning, 8 randomly-selected colonies from the eluted phage titer plates for each library were first analyzed using colony-PCR (as described in Section 1.3) to confirm the presence of VHH inserts. Next, 48 colonies from the round 1 eluted phage titer plates or 96 colonies from the round 2 eluted phage titer plates for each library were sequenced using the M13RP primer. VHH clones of interest were further characterized for their ability to bind recombinant HPV16 E6 using phage ELISA. As similarly described by Baral *et al*. [1] and Hussack *et al*. [2], 1 µg of His6MBP-4C/4S E6, His6MBP-F47R 4C/4S E6, or MBP (100 µL/well; antigens diluted in PBS) or 100 µL/well PBS alone was coated in Nunc™ MaxiSorp™ 96-well plates overnight at 4 °C. The wells were blocked with 5% milk powder in PBS-T at 37 °C, prior to incubation with each respective VHH-displaying phage amplified from the colonies sequenced above for 1 h at 37 °C. The wells were then washed with PBS-T and incubated with a mouse anti-M13 + HRP antibody (GE Healthcare; Mississauga, ON, Canada; Cat. #: 27-9421-01) diluted 1:5 000 in PBS for 1 h at room temperature. The wells were again washed with PBS-T and then incubated with TMB as described above. All clones of interest were tested under these conditions in two separate experiments.

2. Supplementary Data (Tables and Figures)

Supplementary Table 1. Eluted VHHs with the same complementarity-determining region 3 (CDR3) sequences.

Clone	Additional clones with same CDR3 sequence				Total occurrences of each CDR3 sequence
	Library #1, round 1	Library #1, round 2	Library #2, round 1	Library #2, round 2	
A01	-	-	-	-	1
A05	-	-	-	-	1
A09	A13	2A34	-	-	3
A24	-	-	-	-	1
A26	-	-	-	-	1
A27	-	-	-	-	1
A34	-	2A67, 2A69	-	-	3
A37	-	-	-	-	1
A45	-	-	-	-	1
A46	-	-	-	-	1
A47	-	2A05, 2A46	C41	-	4
C06	-	-	-	-	1
C11	-	2A08, 2A13, 2A72, 2A79, 2A89	C22	-	7
C27	-	-	-	-	1
C36	-	-	-	2C37	2
C38	-	2A18, 2A26, 2A33, 2A39	-	-	5
2A03	-	2A37, 2A62	-	-	3
2A04	-	-	-	-	1
2A10	-	-	-	-	1
2A12	-	2A45	-	-	2
2A15	-	-	-	-	1
2A17	-	-	-	-	1
2A51	-	-	-	-	1
2A55	-	-	-	-	1
2A78	-	2A06, 2A48, 2A63, 2A66	-	-	5
2A90	-	-	-	-	1

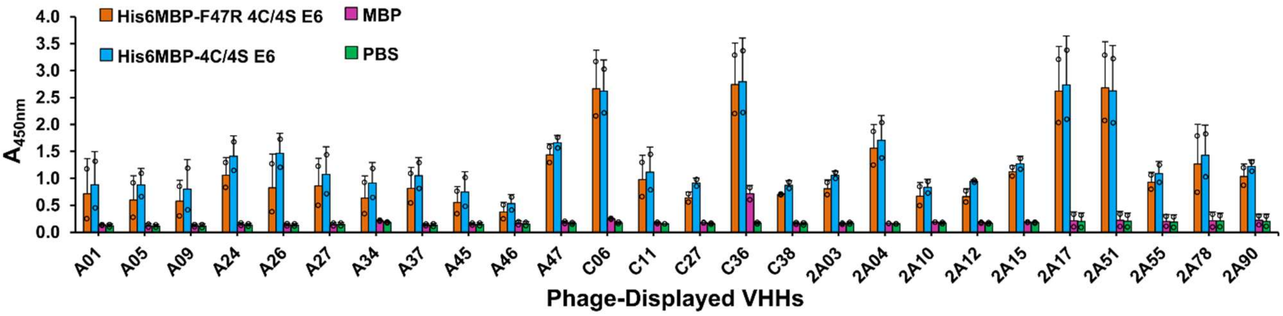

Supplementary Figure 1. Phage ELISA. Clones of interest from the eluted phage titer plates for both libraries were considered to be potential E6 binders if a strong signal was observed following incubation of phage displaying that VHH in wells coated with either His6MBP-F47R 4C/4S-E6 protein but not in wells coated with MBP or PBS. Twenty-six unique clones exhibiting this phage ELISA profile were chosen for soluble expression and purification. Clones beginning with "A" or "C" were isolated from Library #1 or Library #2 round 1 eluted phage titer plates, respectively. Clones beginning with "2A" were isolated from Library #1 round 2 eluted phage titer plates. Data represent mean + standard deviation, with individual data points overlaid (n = 2).

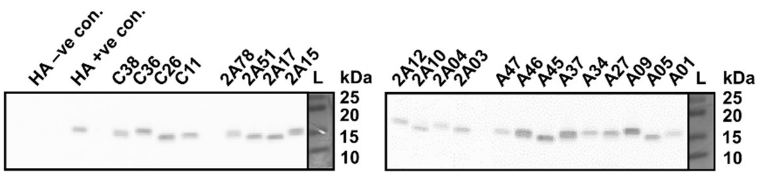

Supplementary Figure 2. Confirmation that the HA tags remained intact on the purified VHHs. Reducing SDS-PAGE Western blots using an anti-HA + HRP antibody demonstrated the HA tags had not been degraded on the purified VHHs which were stored long-term at -20 °C. Approximately 5 ng/lane VHH was loaded. The HA positive (+ve) control is a VHH with both HA and His6 tags which was isolated against an unrelated target antigen. The HA negative (-ve) control is a VHH which alternatively has both Myc and His6 tags.

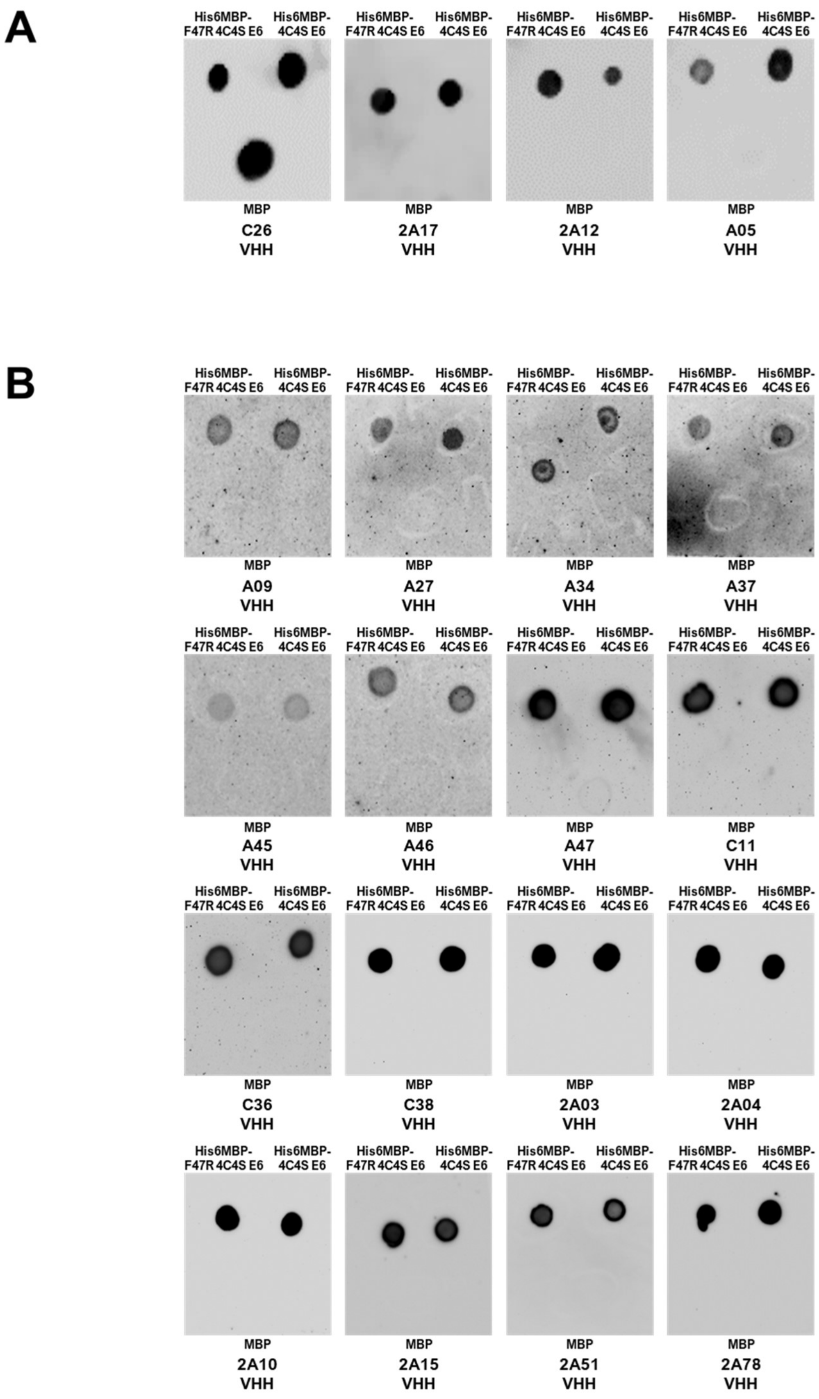

Supplementary Figure 3. (A) Dot blots testing increased VHH concentrations. As observed with the native PAGE Western blots, incubation of the dot blot membranes with 2.7 μg/mL VHHs C26 or 2A17 as the primary antibody yielded a detectable signal. However, 2x this concentration (5.4 μg/mL) and 4x this concentration (10.8 μg/mL) had to be applied to obtain a detectable signal with VHHs 2A12 and A05, respectively. Approximately 2 μg of His6MBP-F47R 4C/4S E6, His6MBP-4C/4S E6, and MBP was spotted on the membrane for each dot. **(B)** The remaining 16/17 VHHs were analyzed using either a concentration of 5.4 μg/mL (A34, C38 and 2A03) or 10.8 μg/mL (A09, A27, A37, A45, A46, A47, C11, C36, 2A04, 2A10, 2A15, 2A51 and 2A78). For one VHH, namely A01, the amount of soluble VHH generated was not sufficient to perform the dot blot assay.

References

1. Baral, T.N.; MacKenzie, R.; Arbab-Ghahroudi, M. Single-domain antibodies and their utility. *Curr. Protoc. Immunol.* **2013**, 2–17. doi:10.1002/0471142735.im0217s103.
2. Hussack, G.; Arbabi-Ghahroudi, M.; MacKenzie, C.R.; Tanha, J. Isolation and characterization of *Clostridium difficile* toxin-specific single-domain antibodies. *Methods Mol. Bio.* **2012**, *911*, 211–239. doi:10.1007/978-1-61779-968-6_14.
3. Hussack, G.; Arbabi-Ghahroudi, M.; van Faassen, H.; Songer, J.G.; Ng, K.K.; MacKenzie, R.; Tanha, J. Neutralization of *Clostridium difficile* toxin A with single-domain antibodies targeting the cell receptor binding domain. *J. Biol. Chem.* **2011**, *286*, 8961–8976. doi:10.1074/jbc.M110.198754.
4. Arbabi-Ghahroudi, M.; MacKenzie, R.; Tanha, J. Selection of non-aggregating V_H binders from synthetic V_H phage-display libraries. *Methods Mol. Biol.* **2009**, *525*, 187–216. doi:10.1007/978-1-59745-554-1_10.
5. Schmitz, U.; Versmold, A.; Kaufmann, P.; Frank, H.G. Phage display: A molecular tool for the generation of antibodies—A review. *Placenta* **2000**, *21*, S106–S112. doi:10.1053/plac.1999.0511.
6. Hussack, G.; Keklikian, A.; Alsughayyir, J.; Hanifi-Moghaddam, P.; Arbabi-Ghahroudi, M.; van Faassen, H.; Hou, S.T.; Sad, S.; MacKenzie, R.; Tanha, J. A V_L single-domain antibody library shows a high-propensity to yield non-aggregating binders. *Protein Eng. Des. Sel.* **2012**, *25*, 313–318. doi:10.1093/protein/gzs014.
7. Pardon, E.; Laeremans, T.; Triest, S.; Rasmussen, S.G.; Wohlkönig, A.; Ruf, A.; Muyldermans, S.; Hol, W.G.; Kobilka, B.K.; Steyaert, J. A general protocol for the generation of Nanobodies for structural biology. *Nat. Protoc.* **2014**, *9*, 674–693. doi:10.1038/nprot.2014.039.
8. Arbabi-Ghahroudi, M.; Desmyter, A.; Wyns, L.; Hamers, R.; Muyldermans, S. Selection and identification of single domain antibody fragments from camel heavy-chain antibodies. *FEBS Lett.* **1997**, *414*, 521–526. doi:10.1016/S0014-5793(97)01062-4.
9. Day, L.A.; Wiseman, R.L. A comparison of DNA packaging in the virions of fd, Xf, and Pf1. In *the Single-Stranded DNA Phages*; Denhardt, D.T., Dressler, D., Ray, D.S., Eds.; Cold Spring Harbor Laboratory: Cold Spring Harbor, NY, USA, **1978**, Volume 8, pp. 605–625. doi:10.1101/087969122.8.605.
